# Reduced DNA methylation and psychopathology following endogenous hypercortisolism – a genome-wide study

**DOI:** 10.1038/srep44445

**Published:** 2017-03-16

**Authors:** Camilla A. M. Glad, Johanna C. Andersson-Assarsson, Peter Berglund, Ragnhildur Bergthorsdottir, Oskar Ragnarsson, Gudmundur Johannsson

**Affiliations:** 1 Department of Internal Medicine and Clinical Nutrition, Institute of Medicine at Sahlgrenska Academy, University of Gothenburg and Department of Endocrinology, Sahlgrenska University Hospital, Gothenburg, Sweden; 2 Department of Molecular and Clinical Medicine, Institute of Medicine at Sahlgrenska Academy, University of Gothenburg, Gothenburg, Sweden; 3 Institute of Neuroscience and Physiology at Sahlgrenska Academy, University of Gothenburg, Gothenburg, Sweden

## Abstract

Patients with Cushing’s Syndrome (CS) in remission were used as a model to test the hypothesis that long-standing excessive cortisol exposure induces changes in DNA methylation that are associated with persisting neuropsychological consequences. Genome-wide DNA methylation was assessed in 48 women with CS in long-term remission (cases) and 16 controls matched for age, gender and education. The Fatigue impact scale and the comprehensive psychopathological rating scale were used to evaluate fatigue, depression and anxiety. Cases had lower average global DNA methylation than controls (81.2% vs 82.7%; *p* = 0.002). Four hundred and sixty-one differentially methylated regions, containing 3,246 probes mapping to 337 genes were identified. After adjustment for age and smoking, 731 probes in 236 genes were associated with psychopathology (fatigue, depression and/or anxiety). Twenty-four gene ontology terms were associated with psychopathology; terms related to retinoic acid receptor signalling were the most common (adjusted *p* = 0.0007). One gene in particular, *COL11A2*, was associated with fatigue following a false discovery rate correction. Our findings indicate that hypomethylation of *FKBP5* and retinoic acid receptor related genes serve a potential mechanistic explanation for long-lasting GC-induced psychopathology.

Hyperactivity of the hypothalamus-pituitary-adrenal (HPA)-axis, with subsequent increase in cortisol exposure at the tissue level^1^,^2^, is implicated in neuropsychiatric disorders such as depression, post-traumatic stress disorder and anxiety^2^,^3^,^4^,^5^,^6^,^7^,^8^,^9^. Cortisol, the predominant glucocorticoid (GC) in humans, affects the central nervous system through binding to its two receptors: the glucocorticoid receptor (GR) and the mineralocorticoid receptor, encoded by the *NR3C1* and *NR3C2* genes, respectively. These receptors are ubiquitously expressed in the brain, particularly in the hippocampus, prefrontal cortex and the parvocellular nucleus of the hypothalamus^10^.

Early-life adverse events have been associated with long-lasting dysregulation of the HPA-axis^11^, which may play a pathophysiological role in development of stress-related diseases^12^,^13^. This early-life molecular programming of the HPA-axis is thought to be conveyed by epigenetic mechanisms^14^,^15^,^16^,^17^,^18^,^19^,^20^. Several studies have shown that *NR3C1* DNA methylation is influenced by both quality of maternal care (rodents)^20^ and experience of childhood trauma (humans)^14^,^16^,^17^,^18^,^19^. Furthermore, increased DNA methylation of the *NR3C1* gene promoter has been observed in the hippocampus and prefrontal cortex in suicide victims with a history of childhood abuse^15^. The mechanism behind this change in DNA methylation is not known, however it is plausible that the increased cortisol exposure induced by psychological stress may be involved.

Marked chronic excess and attenuation of the endogenous diurnal variation in cortisol secretion causes Cushing’s syndrome (CS)^21^, most commonly caused by an ACTH-producing pituitary adenoma (Cushing’s disease; CD) or a cortisol-producing adrenal adenoma. Subjects with CS display a characteristic clinical phenotype including central obesity, muscle and skin atrophy and osteoporosis, as well as marked neuropsychological complaints such as mental fatigue, anxiety, depression and cognitive impairment^22^. Following treatment, most features of the syndrome improve; however, despite long-term remission, we and others have shown that fatigue and cognitive dysfunction commonly persists^23^,^24^,^25^. The mechanism for this persistent cognitive impairment is not known, but the previous excess cortisol exposure is likely to play a mechanistic role^26^. In fact, in our previous study there were no associations between aetiology, treatment (surgery and/or pituitary radiation therapy) or hormone deficiency and cognitive dysfunction^23^.

Due to previous observations of associations between epigenetics and psychopathology^14^,^15^,^16^,^17^,^18^,^19^,^20^, we hypothesized that long-standing excessive cortisol exposure induces changes in DNA methylation that are associated with long-lasting fatigue, depression and anxiety. Here, we used patients with CS as a unique human model of endogenous hypercortisolism to assess the impact of cortisol on genome-wide DNA methylation and its relation to psychopathology.

## Patients and Methods

### Ethical considerations

Informed written consent was obtained from all patients and controls. The local ethical committee of the University of Gothenburg, Sweden, approved the study. The study was conducted according to the Declaration of Helsinki.

### Design

This was a cross-sectional, case-controlled, single centre study including 55 patients with CS in remission and 55 controls matched for age, gender and educational level, as previously described^23^. In this part of the study the association between DNA methylation and fatigue, depression and anxiety in 48 women with CS in remission and 16 controls was analysed. The subjects were studied on three occasions, where medical history was reviewed, physical examination and corticotropin releasing hormone (CRH) stimulation test were performed, blood samples were drawn and psychopathology was evaluated. A 24-h urinary free cortisol (UFC) sampling was performed between the second and last visits, and an overnight dexamethasone suppression test was done following the last visit^23^.

### Patients

The mean age of patients was 53 ± 14 years, and the mean age at diagnosis of CS was 37 ± 14 years (Table 1). Thirty-seven (77%) patients had CD and 11 (23%) had a cortisol producing adrenal adenoma. To verify that the initial diagnosis of CD and cortisol producing adrenal adenoma were correct the clinical, biochemical, radiological and histopathological data from the time of diagnosis were reviewed. In patients with CD in remission the primary treatment was transsphenoidal pituitary surgery in 25 (68%), radiotherapy in five (14%) and bilateral adrenalectomy in seven (19%). Fifteen patients needed additional treatment. In total, 29 (78%) patients with CD were treated with transsphenoidal pituitary surgery, 11 (30%) with radiotherapy and nine (24%) with bilateral adrenalectomy. All patients with cortisol producing adrenal adenoma had been treated with unilateral adrenalectomy. Eighteen (38%) patients had adrenal insufficiency and received replacement therapy with a mean daily hydrocortisone dose of 24 ± 8 mg/day. The mean urinary free cortisol (UFC) excretion was higher in patients compared to controls (Table 1). Seventeen (35%) patients had central (N = 15) or primary (N = 2) hypothyroidism and were receiving a mean L-Thyroxine dose of 104 ± 31 μg/day. Out of 37 patients with CD, 19 (51%) had growth hormone deficiency of whom 15 were on growth hormone replacement therapy. Four out of 20 premenopausal woman (<52 years) had hypogonadotropic hypogonadism and were receiving estrogen and progesterone, 2 of 28 postmenopausal women were receiving treatment with oral estrogen. Two women received replacement with dehydroepiandrosterone.

### Controls

Controls to patients, matched for age and gender, were recruited from a random population sample obtained from the Swedish Tax Agency. Controls were approached through an invitation letter, responding subjects were interviewed per telephone and those who matched the patient’s educational levels and had no previously known psychiatric or chronic diseases known to affect cognitive function, were included. In this part of the study data from one control (N = 16) per three patients (N = 48) were analysed. The mean age was 54 ± 16 years in controls (Table 1).

### Evaluation of hormone status

All patients were in remission, defined by an adequate suppression of serum cortisol concentration (≤50 nmol/l) following a 1 mg overnight dexamethasone suppression test. The median (interquartile range) duration of remission was 13 (5–19) years. A CRH test was performed in order to evaluate the function of the HPA-axis. Serum cortisol was measured using competitive electrochemiluminescence immunoassay (Cortisol Elecsys, Roche Diagnostics Scandinavia AB). Urinary free cortisol (UFC) was measured using radioimmunoassay (SpectRia Cortisol 125I, Orion Diagnostica Oy, Finland). Thyroid function was evaluated clinically and by measurements of free thyroxin and thyroid stimulating hormone (TSH) in serum. Gonadal function was evaluated by asking for menstruation pattern and/or age at menopause as well as measurements of estrogen and gonadotropins in serum. Growth hormone status was evaluated by review of previously performed stimulation tests and measurement of insulin-like growth factor I.

### Evaluation of fatigue, depression and anxiety

Fatigue was evaluated using the fatigue impact scale, a 40 item questionnaire where different aspects of fatigue (physical, cognitive and social) are evaluated^27^. Depression and anxiety were evaluated using the comprehensive psychopathological rating scale^28^.

### DNA isolation and methylation assessment

DNA was isolated from whole blood using the QIAamp DNA Blood Maxi kit (QIAGEN, Hilden, GE). DNA methylation was assessed on the Illumina Infinium HumanMethylation450K BeadChip (Illumina, San Diego, CA, USA), which simultaneously interrogates >465,000 CpG sites and covers 99% of RefSeq genes and 96% of CpG islands. Probes are distributed in CpG islands, shelves, shores, promoter regions, 5′ UTRs, first exon, gene body and 3′ UTRs. Methylation assessment was performed at the Mutation Analysis Facility (MAF) at Karolinska University Hospital. The procedure is briefly described below:

#### Bisulfite treatment

500ng of genomic DNA (OD260/280 > 1.8) was bisulfite treated using the EZ-96 DNA Methylation Kit (D5004; Zymo Research, Inc., Irvine, CA, USA). The CT conversion reagent was mixed with DNA and incubated in the dark at 50 °C for 16 hours. After desulfonation and washing steps, the samples were purified using spin plates, eluted in 12 μl elution buffer and stored at −20 °C prior to processing.

#### Infinium Methylation assay

The Infinium Methylation Assay was performed according to the manufacturer’s instructions. Briefly, 4 μl of denatured bisulfite-treated DNA was isothermally amplified over night at 37 °C, followed by an enzymatic fragmentation step. The fragmented DNA was precipitated, resuspended and loaded (using a Tecan EVO robot) on the 12-sample BeadChip, which was then incubated overnight at 48 °C, allowing the fragmented DNA to hybridize to locus-specific 50-mers. Non-specifically hybridized DNA was washed away, followed by a single-base extension reaction using DNP- and Biotin-labeled ddNTPs (with use of a Tecan EVO robot). Subsequently, hybridized DNA was removed from the labeled oligonucleotide and chips were dried under vacuum and imaged using an Illumina iScan scanner.

### Statistical analyses

#### Clinical parameters

Statistical analyses were performed with IBM SPSS statistics, version 22, or in R version 3.0.3. Data are presented as mean ± standard deviation or median (25–75 percentiles). For comparison between groups we used unpaired t-test for normally distributed data and Mann-Whitney U-test for non-normally distributed data. For proportions, Pearson Chi-square or Fishers exact test were used. Pearson’s correlation was used to determine correlation between methylation and clinical parameters. Linear regression (with adjustment for age and smoking habits) was used to analyse the effect of methylation on clinical parameters.

#### DNA methylation analyses

Data was extracted using GenomeStudio (Illumina, Methylation Module v1.9), which was also used to subtract the background and to normalize staining intensities using internal controls present on the chip. A beta-value was calculated to estimate the methylation level of each CpG locus using the ratio of intensities between methylated and unmethylated alleles (0 = unmethylated, 1 = fully methylated). The performances of the different controls used were evaluated and potential outliers identified. Data quality control and analysis was performed using the ChAMP methylation analysis package (v. 1.4.0)^29^ in R. Briefly, intensity data from IDAT files were loaded, normalized using default settings (i.e. BMIQ) and corrected for batch effects using ComBat. Differentially methylated regions (DMR) were then identified using the Probe Lasso DMR Hunter function with Benjamini-Hochberg p-value adjustment. Correction for multiple testing was done using the “fdrtool” package (v. 1.2.13) in R.

#### Gene ontology analyses

Gene ontology analyses were performed in DAVID Bioinformatics Resources 6.7 (NIAID/NIH) using the Functional Annotation Cluster and Functional Annotation Chart functions^30^,^31^. DAVID provides unadjusted p-values as well as p-values adjusted for multiple testing using both the Bonferroni and the Benjamini methods. Here we present Benjamini-adjusted p-values.

## Results

### Identification of differentially methylated regions and overall DNA methylation

Initial quality control (QC) analyses of the methylation raw data identified one case sample as a technical outlier due to low levels of detected CpG:s (only 38,007 CpG:s were detected with a detection *p*-value < 0.01), this sample was removed from further analysis. The final data set consisted of 47 cases and 16 controls. On average, 485,001 CpG:s were detected (484,979 in cases and 485,066 in controls, detection *p*-value < 0.01).

**Figure 1:**
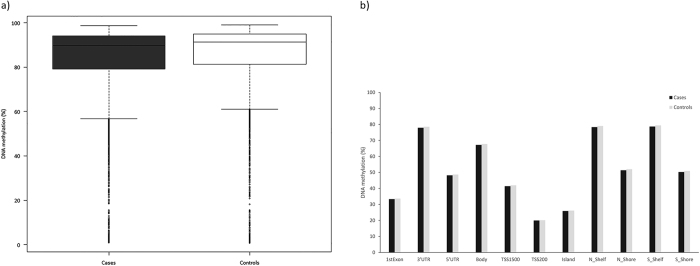
(**a**) Boxplot of average DNA methylation in cases vs controls. (**b**) Bar graph showing DNA methylation in different regions in cases and controls.

We first performed DNA methylation analysis in ChAMP, to assess differences between patients with CS in long-term remission and matched controls (Table 2). Overall, patients had lower average percentage of DNA methylation than controls (81.2% vs 82.7%, *p* = 0.002; Fig. 1a). There were 3,903 probes that lay in differentially methylated regions (DMR:s; n = 461), the majority (n = 3,692; 94.6%) being hypomethylated. Of the 3,903 probes, 3,246 (83.2%) mapped to a gene (n = 337). Of the 337 genes, 278 were exclusively hypomethylated, 7 exclusively hypermethylated and 52 genes contained both hypo- and hypermethylated probes (Supplemental Table 1). Of the 3,903 probes, 55.9% (n = 2,183) had an annotated location; with the most common being gene body (33.3%), 3′-UTR (3.9%) and TSS15 (within 1,500 base pairs upstream or downstream of the transcriptional start site, 2.8%; Fig. 1b).

### Identification of probes associated with fatigue, depression and anxiety

To investigate whether the epigenetic status of the CS subjects is associated with persistent fatigue, anxiety or depression a regression analysis adjusted for age and smoking habits was performed. We identified 731 probes in 236 genes that were associated with at least one of the three clinical traits. Of these 731 probes; 434 were associated with fatigue, 374 with depression, and 452 with anxiety. One hundred and sixty five probes in 108 genes were associated with all three traits.

After multiple testing correction using false discovery rate (FDR; 10%), four probes remained significantly associated with fatigue; cg22890571 (qval: 0.052), cg16479323 (qval: 0.052), cg09502339 (qval: 0.073), and cg07889869 (qval: 0.087). These probes are annotated to the following genes: *TFDP1, ITPK1, COL11A2*, and *DAGLB*. Notably all four probes were also associated with depression and anxiety, however the *p*-values did not remain significant following FDR testing (qval: 0.13–0.23).

### Functional validation of identified probes through gene ontology analyses

**Figure 2:**
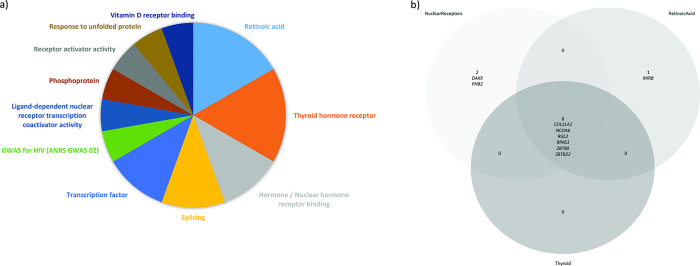
(**a**) Pie chart showing frequency of DAVID terms. Input data: 337 genes, 18 significant DAVID terms after Benjamini correction. These 18 terms are grouped into 11 categories as shown above. Genes included in the GO-term hormone/nuclear hormone receptor binding: *ZBTB22, ZBTB9, PHB2, NCOA6, RING1, COL11A2, DAXX, RGL2.* Retinoic acid: *RXRB, ZBTB22, ZBTB9, NCOA6, RING1, COL11A2, RGL2.* Thyroid hormone receptor: *ZBTB22, ZBTB9, NCOA6, RING1, COL11A2, RGL2*. (**b**) Venn diagram showing overlap of genes included in the three most common GO term families: Retinoic acid (in light grey), Nuclear Receptors (in light blue) and Thyroid (in dark grey). Numbers reflect number of genes in each category.

**Figure 3:**
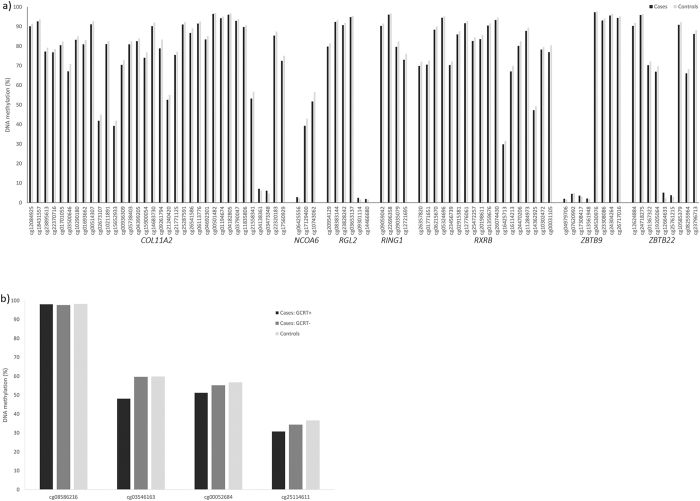
(**a**) Bar graph showing DNA methylation in retinoic acid-related genes in cases and controls. Only probes that were differentially methylated between cases and controls are included. (**b**) Bar graphs showing DNA methylation in the *FKBP5* gene in cases receiving glucocorticoid replacement therapy (black bars), in cases not receiving such therapy (strong grey bars) and in controls (soft grey bars). Only probes that were differentially methylated between cases receiving replacement therapy and cases not receiving such therapy are included.

To explore the functional relevance of the identified DMR:s and clinically associated probes, we next performed gene ontology (GO) analyses using DAVID^30^,^31^. We initially performed GO analysis of all 337 genes with probes that lay in DMR:s and found 202 GO terms, of which 18 were significant after Benjamini correction (Fig. 2a). Terms related to retinoic acid, thyroid hormone receptor and hormone/nuclear hormone receptor binding was the most common (Figs 2b and 3a).

When focusing the analysis on the 236 genes that were associated with at least one of the clinical traits fatigue, depression, and anxiety, 184 terms were identified of which 24 terms were significant after Benjamini correction (Table 3). As before, terms related to retinoic acid were among the most common (Fig. 3a).

### DNA methylation of the GC receptor gene (*NR3C1*)

To explore the potential effect of hypercortisolism on DNA methylation of the *NR3C1* gene, we analysed specifically DNA methylation of this gene. Fifteen out of 49 probes annotated to the *NR3C1* gene were significantly differentially methylated in CS cases compared to controls. After multiple testing correction using false discovery rate (FDR, 10%), all 15 probes remained significant (qval 0.00019–0.065). The most significant difference was observed for probe cg15645634 (*p* = 8.31 × 10^−6^, qval: 0.00019), located in intron 8 of the *NR3C1* gene. Notably, out of these 15 differentially methylated probes, 8 probes were specifically hypermethylated and 7 probes were hypomethylated (Table 4).

### Correlation with markers of HPA-axis activity

To validate the functional value of DNA methylation of the *NR3C1* gene and the genes involved in retinoic acid signalling (in total, n = 672 probes), we performed correlation analyses with urinary free cortisol (UFC; n = 47) and the change in serum cortisol concentration (delta cortisol; n = 24) during a CRH-stimulation test as measures of cortisol exposure and HPA-axis activity, respectively. The CRH-stimulation test was performed in a subgroup of CS patients who had not previously received pituitary irradiation and who did not receive GC replacement therapy. Thirty-one probes were significantly correlated with UFC (Supplemental Table 2), with the strongest correlation observed for probe cg02319187 annotated to the *RXRA* gene (*p* = 0.005; Pearson’s *r* = 0.412). Twenty-five probes were significantly correlated with change in serum cortisol in response to CRH (Supplemental Table 3). The strongest correlation was observed for probe cg00629244, located in the *NR3C1* gene (*p* = 0.002; Pearson’s *r* = 0.598). Three probes (cg01367322, cg03058556 and cg03825390) annotated to the *ZBTB22, ZBTB9* and *RGL2* genes (respectively) were significantly associated with both UFC and the change in serum cortisol. None of the correlations remained significant after correction for multiple testing (FDR, 10%).

### Influence of current GC replacement therapy on DNA methylation

To evaluate the effect of current GC replacement therapy on DNA methylation, we performed a subgroup analysis using the entire dataset, n = 468,149 probes, where patients were stratified by occurrence of current GC replacement therapy. 12,128 probes in 6,186 genes were differentially methylated between the two groups. The most significant differentially methylated probe (cg03546163, *p = *2.99 × 10^−6^; Fig. 3b) was located in the *FKBP5* gene.

In total, there are 34 probes annotated to the *FKBP5* gene on the Illumina 450 K methylation chip and four of these probes showed differential methylation (cg03546163, cg00058684, cg08586216 and cg25114611) in patients with, as compared to without, current GC replacement therapy (Table 5). Most probes annotated to *FKBP5* were hypomethylated in cases receiving GC replacement therapy; (Fig. 3b). No probes remained significantly differentially methylated after multiple testing correction (FDR, 10%). However it is well worth noting that this correction for multiple testing takes into account a very large number of tests, and that in particular the unadjusted *p*-value for *FKBP5* probe cg03546163 reached borderline genome-wide significance (*p = *2.99 × 10^−6^). Together, this suggests a true relevance of this finding, despite qval >0.1.

## Discussion

Hyperactivity of the HPA-axis may increase susceptibility to neuropsychiatric disorders such as depression, post-traumatic stress disorder and anxiety^2^,^3^,^4^,^5^,^6^,^7^,^8^,^9^. Here we provide evidence for a distinguishable pattern of genome-wide DNA methylation in patients previously treated for CS and propose a mechanism for the long-term adverse neuropsychological consequences of endogenous hypercortisolism, which is also commonly observed in a number of different psychiatric disorders.

Aberrations in DNA methylation has been associated with neurological and neuropsychiatric disorders such as autism^32^, schizophrenia^33^ and Alzheimer’s disease^34^, as well as early life adverse events such as child maltreatment and parental stress^17^. Childhood adverse events may influence the life-time set point of the HPA-axis^35^; one plausible mechanism for the induction of a programmed HPA-axis is through GC-induced epigenetic changes. We performed a global DNA methylation analysis to assess differences between patients with CS in long-term remission and matched controls. Generally, patients with CS had lower levels of DNA methylation than controls. Four hundred and sixty-one differentially methylated regions were identified, with the majority being hypomethylated in patients. Also, we identified 731 probes in 236 genes that were associated with at least one of three psychopathological traits; fatigue, anxiety and/or depression. Gene ontology analyses revealed an enrichment of genes functioning as retinoic acid receptors, thyroid hormone receptors or hormone/nuclear hormone receptors. These receptors all belong to the nuclear receptor superfamily^36^ and serve as ligand-activated regulators of gene transcription and stimulators of intracellular pathways. These genes were hypomethylated in cases as compared to controls, and associated with psychopathology in the patients.

The DNA methylation of the genes belonging to the retinoic acid receptor family was also correlated with UFC and change in cortisol concentrations during a CRH-stimulation test, suggesting a functional link between retinoic acid receptor and HPA-axis activity. The retinoic acid family includes Vitamin A and its derivatives, 13-*cis* retinoic acid and all-*trans* retinoic acid. Retinoic acid is a transcriptionally active compound that regulates gene expression via binding to specific nuclear receptors termed RARs^37^, or retinoic X receptors (RXRs)^38^. Retinoic acid is crucial during development of the central nervous system^39^,^40^,^41^ and for neuronal plasticity in adult brain^42^,^43^,^44^. Previous data suggests an involvement of the retinoic acid family in the regulation of the HPA-axis. Both chronic (rats)^45^ and intermittent (humans)^46^ retinoic acid treatment has been shown able to induce HPA-axis hyperactivity and anxiety-like, as well as depressive, behaviour. A plausible mechanism may be that the retinoic acid interrupts the GC receptor induced negative feedback^47^ by down-regulating *11β-HSD1* expression and by inhibiting GC receptor transactivation^48^.

Early adverse events and poor maternal care have been linked to changes in the GC receptor (*NR3C1)* DNA methylation. To explore the potential effect of hypercortisolism on *NR3C1* DNA methylation specifically, we analysed methylation in this gene and found that 15 out of 49 probes annotated to the *NR3C1* gene were differentially methylated in cases as compared to controls. Previously, increased levels of *NR3C1* methylation has been observed in post-mortem hippocampal brain tissue from suicide victims who had endured childhood abuse^15^, and in peripheral blood from subjects with a history of perinatal stress^16^,^17^,^18^ and neglect or abuse during childhood^49^,^50^. Recently, common stressful life events were found to be associated with higher blood DNA methylation of the *NR3C1* gene in adolescents^51^, suggesting that the *NR3C1* DNA methylation is subject to change not only during childhood. In accordance, herein we report that adults who have endured long-term endogenous hypercortisolism have a differential pattern of *NR3C1* DNA methylation than matched controls, lending further support for the importance of excess cortisol exposure as a possible cause in the programming of the HPA-axis and its psychological consequences.

To evaluate the possibly confounding effect of current GC replacement therapy on our results, we performed a subgroup analysis dividing the cases into groups of patients receiving GC replacement or not. These analyses revealed that the DNA methylation of 12,128 probes in 6,186 genes was influenced by current GC replacement therapy. One of the genes that were specifically hypomethylated in cases compared to controls, with an additional reduction in patients currently receiving GC replacement therapy, was the FK506 binding protein 5 (*FKBP5*). FKBP5 binds to and negatively regulates GR function, which subsequently reduces affinity of the GR to cortisol^52^. Common genetic variants in the *FKBP5* gene have been associated with a relative GR resistance, and found to interact with childhood abuse to predict post-traumatic stress disorder^53^. Previously, studies in mice have reported that long-lasting exposure to GC decreases *FKBP5* DNA methylation in the hippocampus, hypothalamus and blood, and that this demethylation is associated with anxiety-like behavior^54^,^55^ and reflect previous GC load^55^. Consistent with these findings, herein we show that *FKBP5* is indeed hypomethylated in CS patients as compared to controls, and that the methylation is further reduced in a sub-group of CS patients receiving GC replacement. These findings validate the suitability of CS as study model for GC exposure and further enlighten the strong effect of GC on DNA methylation.

Despite the rigorous study protocol and adequate study model this project is not without limitations. Firstly, the DNA methylation was assessed in whole blood and not in brain or any other isolated GC target tissue. A recent study, however, provided evidence that DNA methylation variation observed in the brain is in fact reflected in the blood^56^. Secondly, the findings herein remain to be validated and further explored as for whether the observed changes in DNA methylation are indeed associated with subsequent changes in mRNA and protein expression. Lastly, these findings remain to be validated in studies of patients with specific psychiatric disorders with HPA-axis hyperactivity.

In conclusion, our findings suggest that long-standing hypercortisolism reduces global DNA methylation, specifically in genes that are known to attenuate the sensitivity of the GC receptor and therefore may induce hyperactivity of the HPA-axis. Consequently, this may be of importance for the action of cortisol on the central nervous system, and by such contribute to the frequent psychopathology observed in our patients. The mechanism proposed might also apply to other disorders with transient or chronic hyperactivation of the HPA-axis that affects a considerable part of the general population; such as depression, generalised anxiety and post-traumatic stress.

## Additional Information

**How to cite this article**: Glad, C. A. M. *et al*. Reduced DNA methylation and psychopathology following endogenous hypercortisolism – a genome-wide study. *Sci. Rep.* **7**, 44445; doi: 10.1038/srep44445 (2017).

**Publisher's note:** Springer Nature remains neutral with regard to jurisdictional claims in published maps and institutional affiliations.

## Supplementary Material

Supplementary Tables

## Figures and Tables

**Table 1:** Background characteristics, sociodemographic status, psychopathology and hormone measurements in 48 patients with Cushing’s syndrome in remission and 16 controls, matched for age, gender and educational level.

	Patients	Controls	*p*
Age at diagnosis (yr)	37 ± 14	—	—
Age at follow-up (yr)	53 ± 14	54 ± 16	0.9
Duration of remission (yr)	13 (5–19)	—	—
Educational level (%)			1.0
Elementary school	25	25	
Upper secondary education	46	44	
University education	29	31	
Smoking habits (%)			0.8
Non-smoker	53	44	
Ex-smoker	36	44	
Smoker	11	13	
Employment (%)			0.1
Full-time	34	63	
Part-time	30	13	
Sick leave/ Disability pension	11	—	
Retirement	26	25	
Psychopathology			
Fatigue (total score)	63 (40–88)	25 (6–37)	<0.01
Depression (score)	4 (3–7)	2 (1–3)	<0.01
Anxiety (score)	5 (4–7)	3 (3–6)	0.08
Hormone measurements			
S-cortisol – BL (nmol/l)^*^	327 ± 129	305 ± 119	0.6
S-cortisol – Peak (nmol/l)^*^	557 ± 147	584 ± 78	0.5
UFC (nmol/24 h)	202 ± 158	131 ± 59	0.02
FreeT4 (pmol/l)	16.7 ± 3.2	14.8 ± 1.5	< 0.01
IGF-I (μg/l)	149 ± 78	151 ± 84	0.9

Data is presented as mean ± standard deviation or median (interquartile range). ^*^S-cortisol levels were analyzed only in ACTH sufficient patients (N = 30).

S-cortisol was measured in the morning, before (baseline; BL) and after administration of CRH; S-cortisol – peak represents the highest level measured after CRH administration. Psychopathology was evaluated through Fatigue impact scale (FIS) and comprehensive psychopathological rating scale (depression and anxiety).

**Table 2:** Summaries from DNA methylation analyses.

Genes, probes and clinical traits	No
Total no of probes in DMRs	3903
Significantly associated with Anxiety	527 (75 does not match a gene)
Significantly associated with Depression	436 (62 does not match a gene)
Significantly associated with Fatigue	508 (76 does not match a gene)
Total no of probes in genes	3246
Total no of genes with probes	337
No of probes in genes and significantly associated with clinical traits	731
No of genes associated with Anxiety	183
No of genes associated with Depression	172
No of genes associated with Fatigue	194
No of genes associated with Anxiety, Depression and/or Fatigue	236

**Table 3:** DAVID gene ontology analysis with 236 genes with probes significantly associated with at least one clinical trait.

GO-terms	No of genes	Unadjusted *P*-value	Benjamini-adjusted *P*-value
Alternative splicing	135	4.91E-07	0.0002
Genomewide Association Study of an AIDS-Nonprogression Cohort Emphasizes the Role Played by HLA Genes (ANRS Genomewide Association Study 02)	5	3.36E-06	0.0003
GO:0008134~transcription factor binding	23	2.70E-06	0.0006
GO:0004886~retinoid-X receptor activity	5	4.36E-06	0.0007
GO:0042974~retinoic acid receptor binding	6	2.69E-06	0.0012
GO:0030375~thyroid hormone receptor coactivator activity	5	1.12E-05	0.0013
GO:0010861~thyroid hormone receptor activator activity	5	1.12E-05	0.0013
GO:0003708~retinoic acid receptor activity	5	1.66E-05	0.0015
GO:0051059~NF-kappaB binding	6	4.51E-05	0.0034
GO:0030546~receptor activator activity	5	7.55E-05	0.0042
GO:0046966~thyroid hormone receptor binding	6	7.39E-05	0.0047
GO:0006986~response to unfolded protein	9	4.56E-06	0.0062
Splice variant	131	6.79E-06	0.0063
GO:0030374~ligand-dependent nuclear receptor transcription coactivator activity	6	1.32E-04	0.0066
GO:0042809~vitamin D receptor binding	5	2.63E-04	0.0117
GO:0030545~receptor regulator activity	5	7.54E-04	0.0278
GO:0035257~nuclear hormone receptor binding	7	7.06E-04	0.0284
GO:0046978~TAP1 binding	3	1.14E-03	0.0386
GO:0046979~TAP2 binding	3	1.14E-03	0.0386
GO:0046977~TAP binding	3	1.14E-03	0.0386
GO:0042288~MHC class I protein binding	4	1.31E-03	0.0410
GO:0046983~protein dimerization activity	18	1.51E-03	0.0416
GO:0051427~hormone receptor binding	7	1.42E-03	0.0417
GO:0042824~MHC class I peptide loading complex	4	1.65E-04	0.0419

**Table 4:** Methylation in fifteen significantly differentially methylated probes in *NR3C1*.

Probe	Position^a^	Strand	Location	Intron number	Cases^b^	Controls^c^	Delta-beta^d^	*p*	*qval*^e^
cg15645634	142783639	F	Intron	8	0.0356	0.0261	0.0095	*8.31E-06*	*0.000189*
cg14558428	142784982	R	Intron	8	0.0396	0.0314	0.0082	*0.000396*	*0.004514*
cg17860381	142783569	R	Intron	8	0.0412	0.0336	0.0076	*0.00306*	*0.015499*
cg26464411	142784222	R	Intron	8	0.0556	0.0465	0.0091	*0.00406*	*0.017017*
cg18019515	142783385	R	Intron	8	0.0235	0.0192	0.0043	*0.00437*	*0.017388*
cg18146873	142782827	F	Intron	8	0.0279	0.0211	0.0068	*0.00550*	*0.018472*
cg07733851	142781498	R	Intron	8	0.375	0.407	−0.032	*0.00573*	*0.018648*
cg25535999	142757312	R	Intron	7	0.922	0.935	−0.013	*0.0110*	*0.030167*
cg18594054	142623446	R	Upstream		0.926	0.939	−0.013	*0.0144*	*0.035689*
cg04097219	142629749	F	Upstream		0.972	0.978	−0.006	*0.0177*	*0.040303*
cg06770322	142851098	F	Downstream		0.960	0.966	−0.006	*0.0279*	*0.051311*
cg23273257	142658828	R	5′UTR		0.977	0.982	−0.005	*0.0279*	*0.051315*
cg21702128	142784721	F	Intron	8	0.0516	0.0473	0.0043	*0.0306*	*0.053550*
cg25781210	142610141	F	Upstream		0.959	0.967	−0.008	*0.0429*	*0.064421*
cg06521673	142782072	R	Intron	8	0.0227	0.0201	0.0026	*0.0435*	*0.064851*

Methylation in fifteen significantly differentially methylated probes (reported as beta-values) in *NR3C1* on chromosome 5.

^a^Human genome build 37.

^b^CS patients.

^c^Controls.

^d^Difference in methylation between cases and controls.

^e^q-values from multiple correction analysis using a 10% FDR.

**Table 5:** Methylation in *FKBP5*, grouped based on GC replacement therapy.

Probe	Position^a^	GCRT - yes^b^	GCRT - no^c^	Delta_beta^d^	*p*	*qval*^e^
cg03546163	35654363	0.484	0.588	−0.104	2.99E-06	1
cg00052684	35694245	0.514	0.550	−0.036	0.00159	1
cg08586216	35612351	0.981	0.976	0.0050	0.0122	1
cg25114611	35696870	0.314	0.339	−0.025	0.0218	1
cg08915438	35697759	0.559	0.588	−0.029	0.0599	1
cg16052510	35603143	0.809	0.783	0.026	0.0995	1
cg20813374	35657180	0.442	0.462	−0.020	0.109	1
cg00130530	35657202	0.690	0.710	−0.020	0.115	1
cg19226017	35697185	0.752	0.770	−0.018	0.137	1
cg10300814	35565116	0.948	0.953	−0.0050	0.175	1
cg06087101	35551932	0.418	0.440	−0.022	0.197	1
cg14642437	35652521	0.876	0.888	−0.012	0.204	1
cg19014730	35635985	0.681	0.695	−0.014	0.226	1
cg07843056	35656848	0.0257	0.0229	0.0028	0.305	1
cg07485685	35696061	0.0394	0.0375	0.0019	0.409	1
cg17085721	35645341	0.945	0.949	−0.004	0.442	1
cg07061368	35631736	0.894	0.901	−0.007	0.485	1
cg17030679	35696300	0.0215	0.0227	−0.0012	0.520	1
cg00610228	35695934	0.0368	0.0359	0.0009	0.582	1
cg16012111	35656758	0.0484	0.0500	−0.0016	0.585	1
cg23416081	35693573	0.208	0.199	0.009	0.613	1
cg11845071	35695859	0.0209	0.0202	0.0007	0.616	1
cg00140191	35656242	0.0634	0.0615	0.0019	0.661	1
cg08636224	35657921	0.961	0.962	−0.001	0.686	1
cg01294490	35656906	0.0934	0.0913	0.0021	0.687	1
cg18726036	35543610	0.946	0.947	−0.001	0.707	1
cg03591753	35659141	0.539	0.534	0.005	0.720	1
cg14284211	35570224	0.139	0.134	0.005	0.738	1
cg06937024	35695489	0.0259	0.0264	−0.0005	0.791	1
cg00862770	35655764	0.0256	0.0251	0.0005	0.795	1
cg02665568	35544468	0.921	0.923	−0.002	0.799	1
cg07633853	35569471	0.155	0.157	−0.002	0.829	1
cg15929276	35687457	0.187	0.186	0.001	0.911	1
cg10913456	35656590	0.0175	0.0175	0.000	0.989	1

Summary of methylation (reported as beta-values) in *FKBP5* on chromosome 6. GCRT = glucocorticoid replacement therapy.

^a^Human genome build 37.

^b^Patients receiving GCRT.

^c^Patients not receiving GCRT.

^d^Difference in methylation between patients receiving GCRT and those not receiving such therapy.

^e^q-values from multiple correction analysis using a 10% FDR.
