# Supplementary material for: Reduced DNA methylation and psychopathology following endogenous hypercortisolism – a genome-wide study
Source: Sci Rep. 2017 Mar 16;7:44445. doi: 10.1038/srep44445 (PMC5353706; doi:10.1038/srep44445)
Supplement: Supplementary Tables [file srep44445-s1.doc]

**Reduced DNA methylation and psychopathology following endogenous hypercortisolism – a genome-wide study**

Camilla AM Glad, MSc, PhD^1^, Johanna C Andersson-Assarsson, MSc, PhD^2^, Peter Berglund, MD, PhD^3^, Ragnhildur Bergthorsdottir, MD, PhD^1^, ^*^Oskar Ragnarsson, MD, PhD^1^ and ^*^Gudmundur Johannsson, MD, PhD^1^

**SUPPLEMENTAL TABLES**

**Supplemental Table 1.** Summaries of hypo/hypermethylated probes.

| **Hypo/Hypermethylation** | **No** |
| --- | --- |
| No of probes hypomethylated in cases (vs controls) | 3692 |
| No of probes hypermethylated in cases (vs controls) | 209 |
| No of hypomethylated probes in genes | 3081 |
| No of hypermethylated probes in genes | 163 |
| No of genes with hypomethylated probes | 330 |
| No of genes with hypermethylated probes | 59 |
| No of genes with ONLY hypomethylation (number of probes) | 278 (2062) |
| No of genes with ONLY hypermethylation (number of probes) | 7 (29) |
| No of genes with hypo and hypermethylated probes (number of probes) | 52 (1153 [many of them had only 1 hypermethylated probe]) |
|  |  |

**Supplemental Table 2. Probes significantly correlated with urinary free cortisol. Only the ten most significantly associated probes are shown.**

| **Gene** | **Probe ID** | ***p*-value** | **Pearson’s *r*** | **qval^a^** |
| --- | --- | --- | --- | --- |
| *RXRA* | cg02319187 | 0.005 | 0.412 | 1 |
| *COL11A2* | cg15407213 | 0.007 | 0.399 | 1 |
| *NR3C1* | cg27122725 | 0.009 | 0.387 | 1 |
| *RXRG* | cg01913568 | 0.011 | -0.379 | 1 |
| *NR3C1* | cg07528216 | 0.012 | -0.374 | 1 |
| *RGL2* | cg03825390 | 0.012 | 0.374 | 1 |
| *NR3C1* | cg17342132 | 0.021 | 0.346 | 1 |
| *RING1* | cg25026287 | 0.021 | 0.347 | 1 |
| *NR3C1* | cg06952416 | 0.022 | 0.344 | 1 |
| *RGL2* | cg12119313 | 0.025 | 0.337 | 1 |

**a) q-values from correction from multiple testing, 10% FDR.**

**Supplemental Table 3. Probes significantly correlated with delta Cortisol from a CRH-stimulation test. Only the ten most significantly associated probes are shown.**

| **Gene** | **Probe ID** | ***p*-value** | **Pearson’s *r*** | **qval^a)^** |
| --- | --- | --- | --- | --- |
| *NR3C1* | cg00629244 | 0.002 | 0.598 | 1 |
| *RGL2* | cg01606310 | 0.004 | 0.560 | 1 |
| *COL11A2* | cg13586420 | 0.005 | 0.557 | 1 |
| *RGL2* | cg12428738 | 0.006 | 0.542 | 1 |
| *RING1* | cg14046664 | 0.007 | 0.535 | 1 |
| *RXRA* | cg14654324 | 0.009 | 0.520 | 1 |
| *ZBTB9* | cg03058556 | 0.010 | 0.518 | 1 |
| *COL11A2* | cg04770813 | 0.013 | 0.500 | 1 |
| *RGL2* | cg25361447 | 0.015 | -0.492 | 1 |
| *COL11A2* | cg14782559 | 0.016 | 0.484 | 1 |

**a) q-values from correction from multiple testing, 10% FDR.**
